# Single-cell RNA sequencing of human oocytes reveals a differential transcriptomic profile associated with agar-like zona pellucida

**DOI:** 10.1186/s13048-024-01463-8

**Published:** 2024-06-26

**Authors:** Xiangyang Zhang, Senlin Shi, Ying Wan, Wenyan Song, Haixia Jin, Yingpu Sun

**Affiliations:** Reproductive Medical Center, Henan Province Key Laboratory for Reproduction and Genetics, First Affiliated Hospital of Zhengzhou University, Zhengzhou, Henan People’s Republic of China

**Keywords:** Human oocyte, Agar-like zona pellucida, scRNA-seq, Transcriptomic analysis

## Abstract

**Background:**

Agar-like zona pellucida (ZP) is the most common type of abnormal ZP, and is one of the causes of low fertility or infertility. However, the molecular mechanism of agar-like ZP is unclear. Single-cell RNA-sequencing (scRNA-seq) analysis was used to assess the cellular and molecular landscape of oocytes with agar-like ZP.

**Methods:**

Human metaphase I (MI) oocytes were collected from four patients with agar-like ZP and four healthy donors. Total RNA was isolated, cDNA was synthesized, and libraries were generated and subsequently sequenced on a HiSeq 2500 instrument. The scRNA-seq data were analyzed with R software.

**Results:**

We identified 1320 genes that were differentially expressed between agar-like ZP oocytes and healthy donor oocytes. Gene Ontology term enrichment results showed that the genes downregulated in agar-like ZP oocytes were significantly related to extracellular matrix organization, while the genes upregulated in agar-like ZP oocytes were significantly related to the regulation of response to DNA damage stimulus. The Kyoto Encyclopedia of Genes and Genomes enrichment results showed that genes were enriched in the ECM-receptor interaction pathway and focal adhesion pathway. Other signaling pathways important in oocyte development were also enriched, such as PI3K-Akt. Differential expression analysis identified *UBC*, *TLR4*, *RELA*, *ANXA5*, *CAV1*, *KPNA2*, *CCNA2*, *ACTA2*, *FYN* and *ITGB3* as genetic markers of oocytes with agar-like ZP.

**Conclusions:**

Our findings suggest that agar-like ZP oocytes exhibit significant downregulation of genes involved in the ECM-receptor interaction signaling pathway and focal adhesion pathway, which could lead to aberrant ZP formation, while the upregulated genes were significantly related to regulation of the response to DNA damage stimulus. Agar-like ZP formation may interfere with the normal exchange of signals between oocytes and perivitelline granulosa cells, thereby preventing cumulus cells from participating in oocyte DNA damage repair and causing MI arrest.

**Supplementary Information:**

The online version contains supplementary material available at 10.1186/s13048-024-01463-8.

## Background

In humans, oocytes are enveloped by a glycoprotein coat called the zona pellucida (ZP), which contains four glycoproteins (ZP1, ZP2, ZP3, and ZP4) [1]. During oogenesis in mammals, the ZP plays multiple roles, including supporting oocyte growth and promoting the proliferation of follicle cells [2, 3]. In addition, the ZP is involved in regulating oocyte fertilization by allowing only one sperm to penetrate and fuse with the oocyte and preventing the entry of additional sperm [4]. Moreover, the ZP serves to protect both the eggs and developing embryos until they reach the uterus [5]. The ZP also stabilizes the gap junctions or intercellular junctions between oocytes and follicle cells, allowing for the selective passage of certain molecules to growing oocytes [6]. The interactions between granulosa cells and ZP-maintained oocytes during folliculogenesis were shown to be critical for oocyte development in knockout mouse studies [2, 7].

In humans, the ZP proteins are synthesized by oocytes during follicle development [8]. ZP2, ZP3, and ZP4 assemble into heterodimeric repeat units outside the oocyte and are interconnected by ZP1 to form long fibrous filaments [9, 10]. Abnormalities in the ZP are one of the causes of low fertility or infertility, and any structural or functional changes can lead to infertility in females. In mice, homozygous Z*p*2 (Z*p*^−/−^) or Z*p*3 (Z*p*^−/−^) knockout female mice produce ZP-free oocytes that are infertile [2, 11, 12]. Heterozygous Z*p*3 (Z*p*3^+/−^) female mice can produce offspring at a similar rate to normal mice, but the ZP of oocytes from Z*p*3 (Z*p*3^+/−^) mice is thinner than that of oocytes from nonmutant mice [13]. Homozygous Z*p*2 (Z*p*^−/−^) knockout female mice produce oocytes with a looser ZP-looser, which is composed of ZP2 and ZP3 around the oocyte [7]. Various ZP abnormalities, including an absent ZP [14], an irregularly shaped ZP, a dark ZP [15], a thin ZP [16, 17] and an agar-like ZP [18, 19], have been reported in humans. Agar-like ZP is the more common ZP abnormality, and mainly manifests as a clear, dense ZP and a completely or partially absent perivitelline space. To date, no gene mutations related to agar-like ZP have been identified, and only a few cases have been reported.

In this study, we describe four patients who were diagnosed with primary infertility. Conventional in vitro fertilization (IVF) failed in these patients due to ZP abnormalities in the oocytes. Furthermore, during each IVF cycle, almost all the oocytes with abnormal ZP did not undergo first polar body expulsion. Using single-cell sequencing analyses, we conducted a comprehensive investigation of the transcriptional profiles of human oocytes exhibiting agar-like ZP and compared them to those of normal oocytes. Single-cell RNA sequencing(scRNA-seq) analyses provide an opportunity to discover the mechanisms underlying agar-like ZP. This study revealed a difference in mRNA storage between agar-like ZP oocytes and normal ZP oocytes and revealed a possible molecular mechanism of metaphase I (MI) arrest in agar-like ZP oocytes.

## Results

### Clinical characterization of individuals with oocytes with agar-like ZP

Four unrelated individuals (T1, T2, T3, and T4) are described in this study (Table 1). The patients enrolled in the study had a history of infertility for several years and had 2 to 4 failed IVF treatment cycles.

**Table 1:** Primary physiologic indices of patients

patient	Maternal age	BMI	cycle numbers	Stimulation protocol	Basal FSH (mIU/ml)	Basal E2 (pg/ml)	Basal *P* (ng/ml)	Basal LH (mIU/ml)	Number of retrieved oocytes	Number of mature oocytes
C1	34	21.5	1	GnRH-a long	5,84	44.13	0.75	5.76	24	21
C2	30	23	1	GnRH-a long	5.83	33.94	0.24	5.54	17	15
C3	32	22	1	GnRH-a long	5.62	55.39	0.32	3.25	13	11
C4	31	24.3	1	GnRH-a long	8.41	31.12	0.13	5.99	12	10
T1	35	21.9	2	GnRH-a long/ GnRH-a super-long	7.61	22.02	0.49	5.22	26	2
T2	24	23.88	4	GnRH-a long/ GnRH-a super-long	4.67	36.4	0.35	5.42	34	0
T3	26	25.5	2	GnRH-a long/ GnRH-a super-long	6.07	17.52	0.46	6.3	28	0
T4	32	19.2	2	GnRH-a long/ GnRH-a super-long	6.21	38.38	0.6	6	31	1

**Fig. 1:**
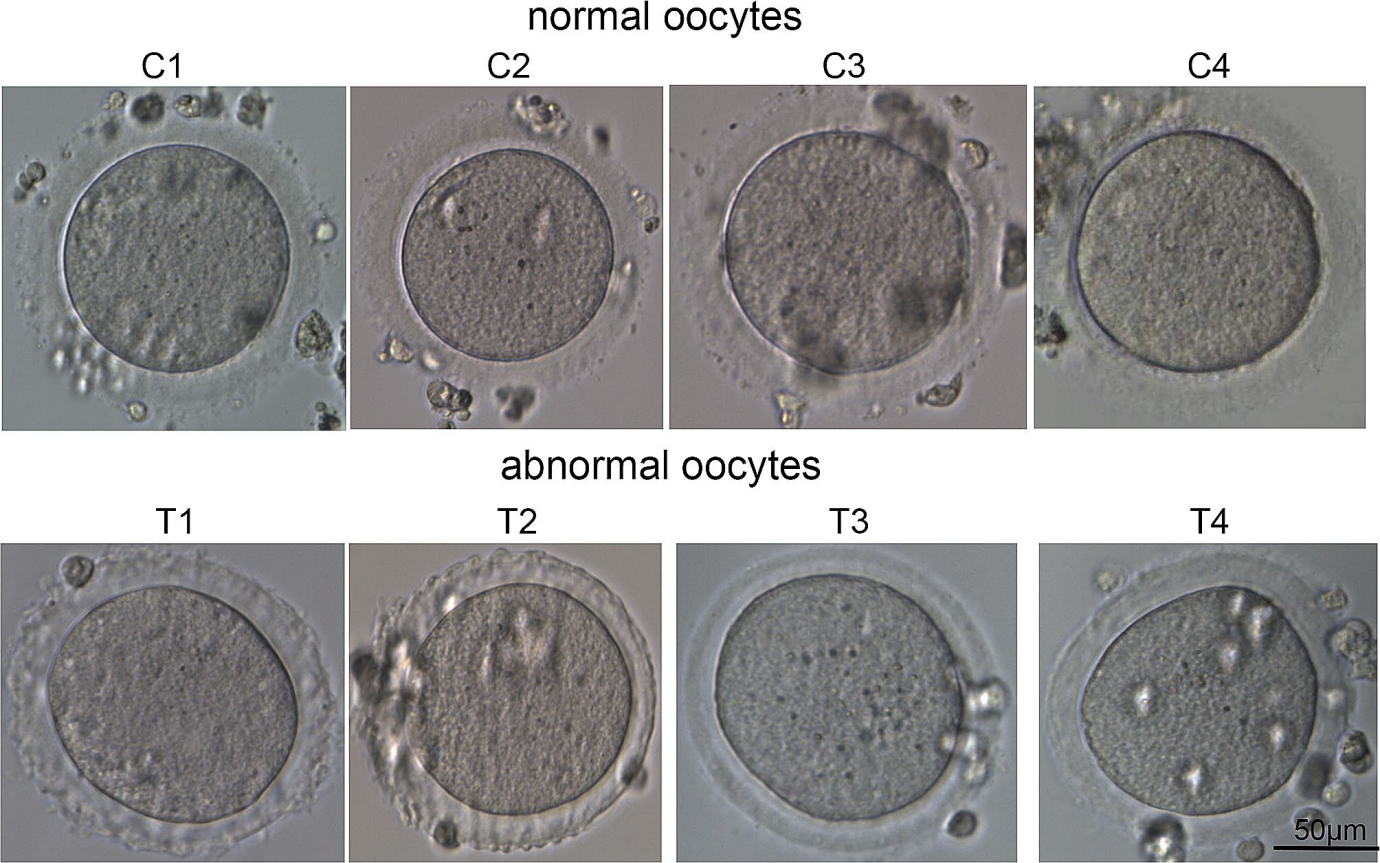
Features of agar-like ZP. The ZP was translucent and dense or had irregular protrusions under an inverted microscope, showing agar-like structures with a total or partial absence of the perivitelline space

After the granulosa cells were removed, the ZP of the oocytes from these patients was observed to be translucent and dense or to have irregular protrusions under an inverted microscope, revealing agar-like structures with a total or partial absence of perivitelline space (Fig. 1). The oocytes of these patients were arrested mainly at the MI stage.

### Transcriptional profiles of single human normal and abnormal oocytes

With ethical approval, we obtained a total of 4 MI oocytes from 4 healthy egg donors and 4 MI oocytes from 4 patients with agar-like ZP. The physiological indices of the patients in our study did not differ, and similar IVF procedures were used for all patients. The agar-like ZP oocytes were arrested at the MI stage, while their morphologies and sizes are similar to those of normal ZP MI oocytes. Our study analyzed 4 normal MI oocytes (normal) and 4 agar-like ZP MI oocytes (abnormal) and generated eight individual high-quality scRNA-seq datasets. Approximately 40 million reads were detected for each sample, for a clean read rate greater than 91.9%. An average of 97% of the reads mapped to the human genome, and more than 60% constituted exon reads, indicating high coverage.

**Fig. 2:**
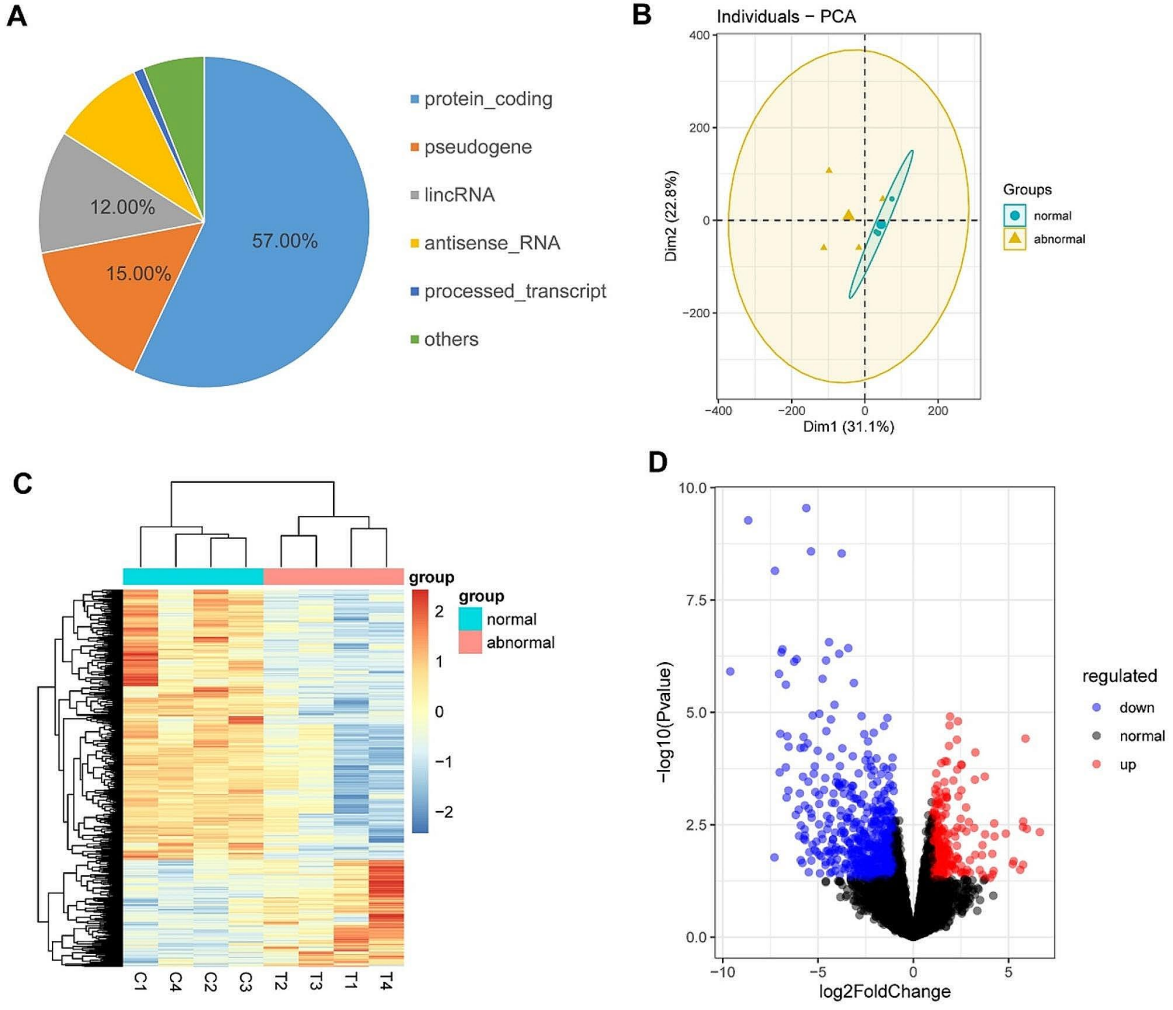
Characteristics of and differences between transcriptomes from normal and abnormal human oocytes. (A) The majority of the genes were protein-coding genes. (B) Principal component analysis (PCA). (C) Heatmap of differentially expressed genes in each comparison group. The genes with P value < 0.05 and |log_2_ fold change|≥1 according to differential expression analysis were extracted, and heatmaps were generated to visualize the expression of each sample. Blue indicates a lower expression level, and red indicates a higher expression level. (D) Volcano map of differentially expressed genes

A total of 30,446 transcripts were expressed, with protein-coding genes being the most annotated (53.7%), followed by long noncoding RNA (lncRNA) genes (27.4%) and pseudogenes (14.4%) (Fig. 2A). To further explore the differences between normal and abnormal samples, we clustered genes using principal component analysis (PCA). PCA was carried out on the obtained RNA-seq normalized data (log fragments per kilobase of transcript per million mapped reads (FPKM) using an unsupervised method. A significant difference was observed in the clustering of MI oocytes according to the RNA-seq data between the healthy and abnormal groups (Fig. 2B). In the PCA plot, the normal samples were clustered together, with small differences. However, the abnormal group was relatively scattered, indicating that there were large differences between oocytes in the abnormal group. We construted a heatmap of the differentially expressed genes (DEGs) in the 8 samples (Fig. 2C). A total of 1320 genes were considered DEGs (|log_2_ fold change|≥1 and P value < 0.05). We identified 938 genes that were significantly downregulated and 382 genes that were upregulated, of which 648 and 278 genes, respectively, were protein-coding genes, (Fig. 2D).

### Expression of ZP genes and oocyte maturation arrest-related genes

**Fig. 3:**
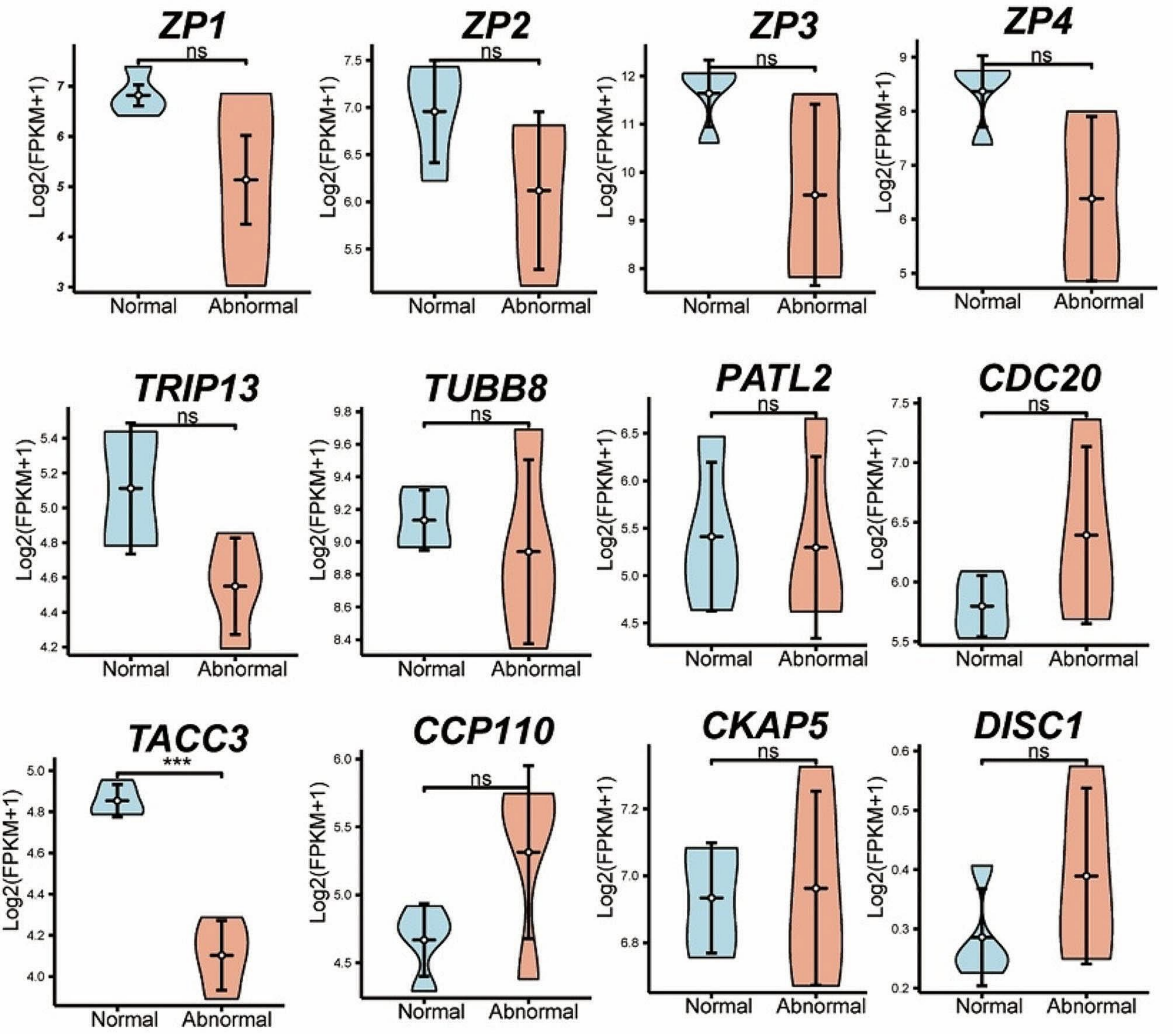
Violin plots showing the relative expression levels (log_2_ [FPKM + 1]) of ZP genes and oocyte maturation defect genes between normal and abnormal oocytes. A P value < 0.05 was considered to indicate statistical significance and is indicated with an asterisk (*). FPKM: fragments per kilobase per million

ZP gene mutations can cause abnormalities in ZP structure. Next, We analyzed the expression of four ZP genes (ZP1, ZP2, ZP3, and ZP4), and there was no significant difference between the normal and abnormal groups (Fig. 3). Patients with agar-like ZP produced oocytes that were mostly arrested in the MI -stage. We analyzed the reported oocyte maturation arrest genes *TUBB8*, *TRIP13*, *PATL2*, *CDC20* and *TACC3* (Fig. 3). The expression levels of *TUBB8*, *TRIP13*, *PATL2* and *CDC20* expression levels did not differ between the normal and abnormal groups. Compared to those in the control group, the expression of the *TACC3* gene was significantly lower in the abnormal group(*P* < 0.001). The human oocyte microtubule organizing center (huoMTOC) protein TACC3 is responsible for microtubule polymerization and plays a critical role in cell spindle assembly. In addition to TACC3, CCP110, CKAP5 and DISC1 are huoMTOC proteins. Therefore, we also analyzed the expression of the *CCP110*, *CKAP5* and *DISC1* genes. The expression levels of *CCP110*, *CKAP5* and *DISC1* did not differ between the normal and abnormal groups (Fig. 3).

### GO term and KEGG pathway enrichment analyses of the DEGs

**Fig. 4:**
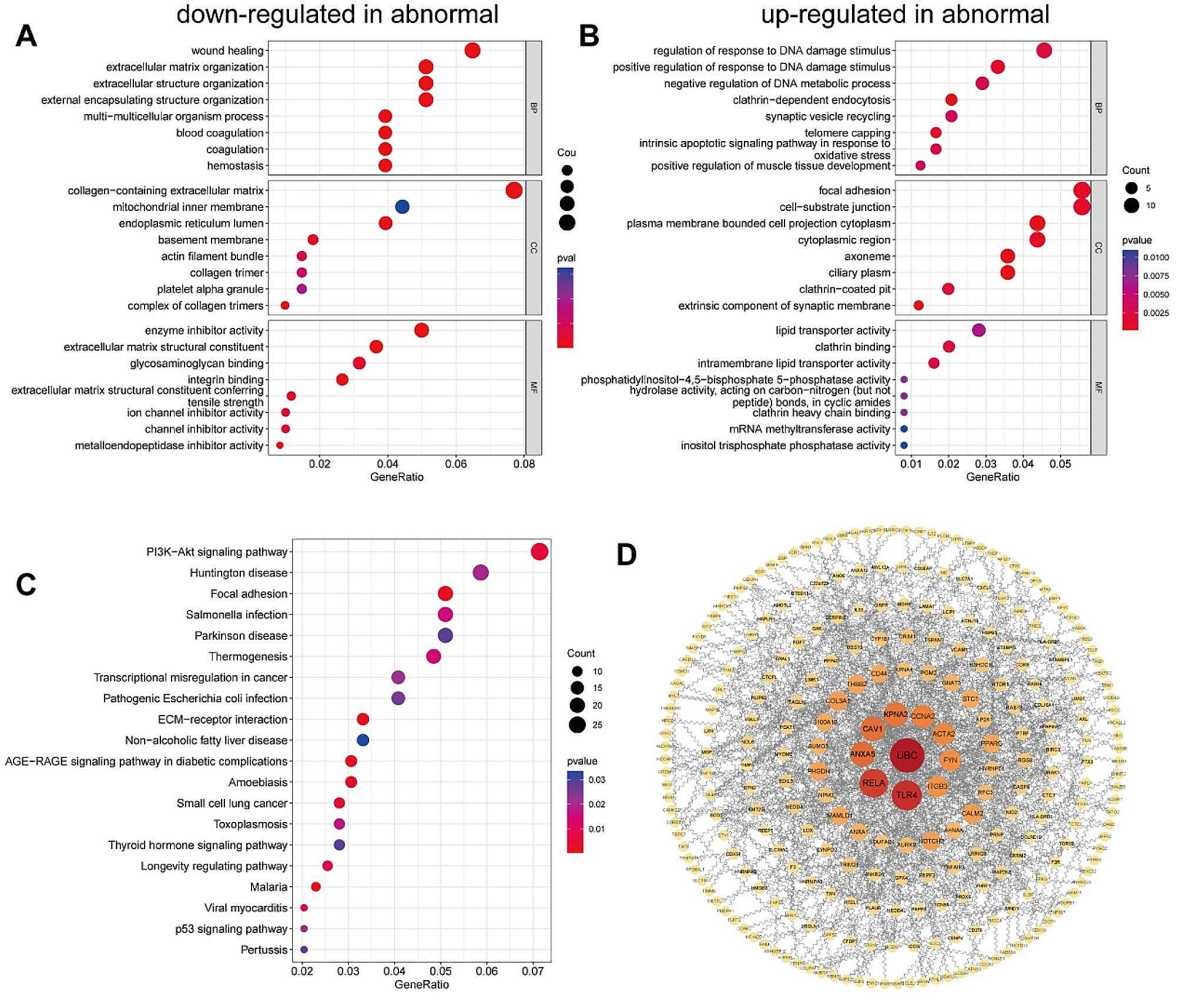
GO term enrichment, KEGG pathway analysis and PPI analysis of DEGs. (A) Gene Ontology analysis of genes downregulated in abnormal oocytes. (B) Gene Ontology analysis of genes upregulated in abnormal oocytes. (C) The 20 most significantly enriched pathway terms (upregulated and downregulated). The dot size represents the number of genes annotated with the indicated GO and KEGG terms; the color represents the adjusted -*p* value of the terms. (D) The PPI network of DEGs between normal and abnormal tissues. The octagonal nodes represent DEGs, and the edges represent interactions between nodes. The PPI network was generated using STRING and visualized in Cytoscape

To investigate the potential effects of the DEGs, we performed GO analysis on the DEGs identified through DESeq2. We identified GO terms enriched for down- and up-regulated genes. The downregulated genes, were most enriched in the GO terms extracellular structure organization, integrin binding and extracellular matrix (EMC) organization (Fig. 3A). The upregulated genes, were most enriched in the biological process regulation of the response to DNA damage stimulus (Fig. 3B). These results strongly suggested that the genes downregulated in oocytes with agar-like ZP were closely related to the ECM, while the upregulated genes strongly correlated with the DNA damage response (DDR), suggesting that abnormal ECM and DDR functions are likely to be important reasons underlying the differences between normal and agar-like ZP oocytes. The relevant DEGs of DDR included *NSMCE4A*, *FXR2*, *TRIM28*, *ARMT1, MMS19, CCAR2, INO80E, POLH, HIC1, TRRAP, RTEL1*. KEGG database analysis was used to identify the potential signaling pathways associated with the DEGs. Twenty-four pathways in which both upregulated and downregulated genes were enriched were identified; the 20 pathways related to the gene exhibiting the most significant enrichment are shown in Fig. 4C. The enrichment results showed that the largest number of affected genes in oocytes with abnormal agar-like ZP were associated with the ECM-receptor interaction pathway, focal adhesion pathway and PI3K-Akt signaling pathway.

To identify key candidate genes, we analyzed protein-protein interaction (PPI) networks of 403 protein-coding DEGs (|log_2_ fold change|≥1 and P value < 0.01) using the STRING online database and Cytoscape software. Among them, 269 nodes and 642 edges were included in the DEG PPI network (Fig. 4D). After constructing the PPI network, ten genes with high connectivity, namely, *UBC, TLR4, RELA, ANXA5, CAV1, KPNA2, CCNA2, ACTA2, FYN* and *ITGB3*, were selected as hub genes.

## Discussion

In this study, we conducted a transcriptome analysis of human MI oocytes with normal ZP and agar-like abnormal ZP. The DEGs with low expression in abnormal agar-like ZP oocytes were enriched mainly in ECM structure-related pathways, while the DEGs with high expression were enriched mainly in DNA damage response pathways.

Abnormalities in the ZP in oocytes are mostly related to mutations in ZP genes. In humans, a frameshift mutation (I390fs404X) in *ZP1* has been reported to produce a truncated ZP1 protein, resulting in the absence of a ZP in oocytes [14]. The *ZP1* homozygous missense mutations p. Val570Met and p. Arg410Trp do not affect the expression of the ZP1 protein but affect the secretion of the ZP1 protein to the exterior of the cell, resulting in cumulus–oocyte complexes lacking eggs or eggs without a ZP [20]. The *ZP2* homozygous missense mutation p. Cys372Ser does not affect the expression of the ZP2 protein but affects its secretion to the exterior of the cell, resulting in eggs without a ZP or mature eggs with a thin ZP, in addition, mature eggs exhibit poor embryonic development after fertilization [20]. These results suggest that the mutated ZP protein is blocked from being secreted and fails to assemble in vivo. The *ZP3* heterozygous missense mutation p. Arg255Gly does not affect the secretion of the protein, but the expression of the ZP3^R255G^ protein is significantly elevated, which resulted in altered expression of other cotransfected ZP proteins. Resulting in oocytes without a ZP [20]. According to a recent study, individuals with a thin and irregular ZP were found to have a heterozygous mutation in the *ZP4* gene, resulting in reduced ZP4 secretion [21]. Other studies have reported ZP gene mutations causing thinning of the ZP [22]. In summary, in humans, mutations in ZP genes cause abnormal expression or secretion of ZP proteins, which in turn affects the assembly of the ZP matrix, resulting in the absence or thinning of the ZP in oocytes. However, to date, no ZP gene mutation has been found to be associated with agar-like ZP.

Yang D et al. reported that the agar-like ZP of oocytes is not affected by external factors such as ovulation induction scheme, operation time, age and hormones but may be related to individual patient factors [19]. Sousa et al. described a patient in whom the ZP morphology was abnormal, with an external indentation and a reduced or absent perivitelline space, similar to that observed in the T2 sample of our study. Transmission electron microscopy (TEM) of those oocytes revealed numerous empty spaces within the ZP, indicating a loose structure [23], which suggested that the agar-like ZP had an abnormal structure. There was no significant difference in ZP gene expression between normal and abnormal oocytes, but ZP protein expression was not detected because the samples were very rare. scRNA-seq data revealed that the gene expression profiles of agar-like ZP oocytes were markedly different from those of normal oocytes. The most affected genes in agar-like ZP oocytes clustered in similar GO terms, such as those relate to the extracellular matrix. Target gene enrichment analysis revealed that the terms in which the DEGs wer enriched were related to the focal adhesion pathway and the ECM-receptor interaction pathway, which are involved in mutual signaling between oocytes and follicular cells [24, 25]. By constructing a PPI network, we identified ten highly connected hub genes, *UBC*, *TLR4*, *RELA*, *ANXA5*, *CAV1*, *KPNA2*, *CCNA2*, *ACTA2*, *FYN* and *ITGB3*, that may be associated with the synthesis or formation of ZP proteins.

In addition to the presence of agar-like ZP, oocyte maturity defects, particularly MI stage arrest, were also observed. Compared with that in normal controls, the expression of genes related to the DNA damage response was upregulate in agar-like ZP oocytes, suggesting the occurrence of DNA damage. Immature oocytes possess low efficiency DNA damage response mechanisms and are therefore largely ineffective at DNA repair [26, 27]. In contrast, GV-stage oocytes can resume meiosis even with widespread DNA damage and are able to progress into MI [26, 27]. The DDR is a cellular mechanism that functions to repair DNA damage [28]. DNA damage can be categorized as programmed or spontaneous. There are different types of DNA damage, such as DNA mismatches, DNA single-strand breaks (SSBs), double-strand breaks (DSBs) and so on [28]. The cause of the agar-like ZP oocyte DNA damage response is unclear. The physiological process of gametogenesis involves the formation of DSBs through meiotic recombination. Meiotic recombination occurs via the formation and subsequent repair of DSBs. Fully grown mouse oocytes are less sensitive to DSBs [29]. Evidence has shown that oocytes cooperate with the surrounding cumulus cells to regulate the DDR [30]. These finding suggested that cumulus cells are involved in oocyte DDR. In clinical practice, compared to those of normal controls, fewer cumulus cells surround oocytes with agar-like ZP. There may be interference in the communication between oocytes and cumulus cells due to the presence of agar-like ZP, which affects the participation of cumulus cells in oocyte DDR.

MI arrest in oocytes duo to DNA damage is triggered by SAC activation [26, 31, 32]. SAC is activated when chromosomes are mislocalized or fail to attach to the spindle microtubules; SAC components localize to the kinetochore, where they repress APC/C [32]. Our investigation demonstrated a marked decrease in the expression of *TACC3* in oocytes with agar-like ZP. TACC3 is a huoMTO protein in human oocytes. MTOC proteins are responsible for polymerizing microtubules and are crucial for spindle assembly. Studies have shown that certain compound heterozygous mutations in *TACC3* cause a loss of function, resulting in oocyte maturation arrest [33]. Another study demonstrated that overexpression of *TACC3* disrupted the DDR in normal cells and resulted in defective checkpoint and DSB-mediated homologous recombination (HR) repair systems, leading to genome instability [34]. We speculate that the downregulation of *TACC3* expression in oocytes with an abnormal ZP is the result of negative feedback from the DDR.

## Conclusions

The ZP is crucial for the development of oocytes and follicles during oogenesis. Oocytes with agar-like ZP displayed distinctive gene expression profiles compared to those with a normal ZP. Our findings suggest the significant downregulation of genes involved in the focal adhesion and ECM-receptor interaction signaling pathways, which could lead to aberrant ZP formation. Agar-like ZP formation may interfere with the normal exchange of signals between oocytes and perivitelline granulosa cells, thereby preventing cumulus cells from participating in the oocyte DDR and causing MI arrest.

## Methods

### Human oocyte collection

Ethical approval for this study was obtained from the Institutional Review Board (IRB) of The First Affiliated Hospital of Zhengzhou University (2020-KY-186). In our study, all the oocytes used for research purposes were voluntarily donated by IVF patients who were fully informed of the goals and objectives of our experiments. All research was performed in accordance with relevant guidelines/regulations and in accordance with the Declaration of Helsinki (2013). All patients included in this study signed a written informed consent.

The abnormal group consisted of agar-like ZP oocytes (T1, T2, T3, and T4) obtained from patients whose oocytes had abnormal ZP and had not fully matured. The control group consisted of normal oocytes (C1, C2, C3 and C4) collected from individual IVF patients who produced MII oocytes exhibiting normal maturation rates; MI oocytes which are usually discarded, were voluntarily donated to our study to analyze gene expression patterns, and MII oocytes were used for IVF treatment (Table 1).

The same procedures were used for the abnormal and normal groups, including clinical and oocyte processing. Following human chorionic gonadotropin (hCG) administration, ovarian stimulation was performed, and oocyte collection using transvaginal ultrasound guidance was scheduled 36 h after hCG administration. Mature MII oocytes from normal patients were used for IVF treatment, and the remaining MI oocytes were cryopreserved. For the abnormal group, immature oocytes at the MI stage were recovered after 24 h of culture and cryopreserved. Normal and abnormal MI oocytes were ultimately collected and cryopreserved.

### Vitrification/thawing

All tools and materials for vitrification and thawing were obtained from Kitazato (Japan). The cryotop protocol was used for oocyte vitrification. Oocytes underwent a stepwise equilibration procedure in ES for 12 min, after which they were treated with a vitrification solution for 40–60 s. The oocytes were placed in a minimal volume of VS, and the cryotop was directly immersed in liquid nitrogen for rapid cooling. During warming, the cryotop sheet underwent a rapid 1-minute immersion in TS at 37 °C followed by a 3-minute dilution step in DS at room temperature. After dilution, two washes in WS were performed on the samples, with the first wash lasting 5 min and the second lasting 3 min, both at room temperature.

### cDNA synthesis, library preparation, and sequencing

Single-cell transcriptome sequencing of 8 human oocytes, including 4 abnormal and 4 normal oocytes, was conducted by Annoroad Gene Technology Co., Ltd. (Beijing, China). Oocytes were collected in tubes with lysis buffer and ribonuclease inhibitor to prevent RNA degradation. The Smart-Seq2 method was used to amplify the collected samples. To enrich the cDNA, PCR amplification, which was performed using reverse transcription with an oligo-dT primer, was carried out after first-strand cDNA synthesis. The amplified cDNA was then purified using MagBeads. After cDNA production, we evaluated the yield and quality of the cDNA using both a Qubit^®^ 3.0 fluorometer and an Agilent 2100 bioanalyzer to confirm that the cDNA fragments were in the expected size range of 1–2 kilobases. Next, ultrasonic waves were used to randomly shear the cDNA to generate fragments suitable for the Illumina library preparation protocol. The process involved DNA fragmentation, end repair, A-tailing at the 3’ ends, ligation of adapters, PCR amplification, and library validation for high-quality sequencing. Once the libraries were prepared, we utilized quality control methods to ensure high-quality sequencing results. Library quality was checked using the PerkinElmer LabChip^®^ GX Touch and Step OnePlus™ Real-Time PCR System. The Illumina HiSeq platform was used to sequence the libraries with a paired-end read length of 150 bp after passing quality control checks.

### Single-cell transcriptome analysis: expression profiling and pathway enrichment

HISAT2 (version 2.1.0) with default settings was used to map all reads to the human genome (version GRCh38.99.chr). The HTseq tool (version 0.6.0) was used to calculate the FPKM values. Genes with FPKM values greater than or equal to 3 were classified as expressed. In subsequent analyses, noncoding genes were identified using the biotype classification of genes and transcripts from the ENSEMBL annotation. For differential expression analysis, to estimate the expression levels of all isoforms of a gene, we used DESeq2 (version 1.20.0) for DEG analysis, considering genes with a P value < 0.05 and |log_2_ fold change| ≥ 2 as DEGs, and visualized the DEGs through the use of a volcano plot. To annotate and enrich the DEGs, we performed a two-sided Fisher exact probability test to classify GO categories and carried out KEGG pathway enrichment analysis. *P* < 0.05 was considered to indicate statistical significance for the GO terms or KEGG pathways.

### Statistical analyses

The data were analyzed and compared using R4.2.2 software. A two-group comparison of the gene expression data between the two groups was performed. A P value of less than 0.05 was considered to indicate statistical significance and is denoted by an asterisk in the figures.

### Electronic supplementary material

Below is the link to the electronic supplementary material.

Supplementary Material 1

## Data Availability

The raw sequence data reported in this paper have been deposited in the Genome Sequence Archive (Genomics, Proteomics & Bioinformatics 2021) in National Genomics Data Center (Nucleic Acids Res 2022), China National Center for Bioinformation / Beijing Institute of Genomics, Chinese Academy of Sciences (GSA-Human: HRA004898) that are publicly accessible at https://ngdc.cncb.ac.cn/gsa-human.
